# Coronavirus (COVID-19): A Review of Clinical Features, Diagnosis, and Treatment

**DOI:** 10.7759/cureus.7355

**Published:** 2020-03-21

**Authors:** Syed Adeel Hassan, Fahad N Sheikh, Somia Jamal, Jude K Ezeh, Ali Akhtar

**Affiliations:** 1 Internal Medicine, Dow University of Health Sciences, Karachi, PAK; 2 Pathology, Sahiwal Medical College, Sahiwal, PAK; 3 Internal Medicine, Abbasi Shaheed Hospital, Karachi, PAK; 4 Internal Medicine, Newark Beth Israel Medical Center, Jersey City, USA; 5 Internal Medicine, Army Medical College, National University of Medical Sciences, Rawalpindi, PAK

**Keywords:** cough, dyspnea, rna virus, pathogenesis, clinical features, zoonoses, covid-19, sars-cov-2, coronavirus, diagnosis

## Abstract

Coronavirus (COVID-19) is an enveloped RNA virus that is diversely found in humans and wildlife. A total of six species have been identified to cause disease in humans. They are known to infect the neurological, respiratory, enteric, and hepatic systems. The past few decades have seen endemic outbreaks in the form of Middle East respiratory syndrome coronavirus (MERS-CoV) and severe acute respiratory syndrome related coronavirus (SARS-CoV). Yet again, we see the emergence of another outbreak due to a new strain called the SARS-CoV-2 virus. The most recent outbreak initially presented as pneumonia of unknown etiology in a cluster of patients in Wuhan, China. The epicenter of infection was linked to seafood and exotic animal wholesale markets in the city. SARS-CoV-2 is highly contagious and has resulted in a rapid pandemic of COVID-19. As the number of cases continues to rise, it is clear that these viruses pose a threat to public health. This review will introduce a general overview of coronavirus and describe the clinical features, evaluation, and treatment of COVID-19 patients. It will also provide a means to raise awareness among primary and secondary healthcare providers during the current pandemic. Furthermore, our review focuses on the most up-to-date clinical information for the effective management, prevention, and counseling of patients worldwide.

## Introduction and background

Coronavirus (CoV) is a large family of positive-sense, single-stranded RNA viruses that belong to the Nidovirales order. The order includes Roniviridae, Arteriviridae, and Coronaviridae families [1]. The Coronaviridae family is subdivided into Torovirinae and Coronavirinae subfamilies [1]. Coronavirinae is further subclassified into alpha-, beta-, gamma-, and delta-COVs [1]. Phylogenetic clustering accounts for the classification of these subtypes of viruses. Their viral RNA genome ranges from 26 to 32 kilobases in length [2]. They can be isolated from different animal species. These include birds, livestock, and mammals such as camels, bats, masked palm civets, mice, dogs, and cats [2]. The widespread distribution and infectivity of COV make it an important pathogen.

Human pathogenic subtypes of CoV are associated with mild clinical symptoms. However, severe acute respiratory syndrome related coronavirus (SARS-CoV) and Middle East respiratory syndrome coronavirus (MERS-CoV) are the two notable exceptions. In 2012, MERS-CoV was first detected in Saudia Arabia. It was responsible for 2,494 confirmed cases, which led to 858 fatalities [2]. In 2002, a subtype of the beta-COV rapidly spread across Guangdong, China. This outbreak resulted in 8,000 infections and 774 fatalities in 37 countries [2]. The outbreak in 2020 has presented in the form of pneumonia of unknown etiology in Wuhan, China. Deep sequencing studies and lab investigations have identified the culprit as a new strain of COV [3]. Initially, this virus was designated as 2019-nCoV. However, the International Committee on Taxonomy of Viruses designated it as the SARS-CoV-2 virus [4]. On February 11, 2020, the World Health Organization (WHO) announced the disease caused by this novel virus as coronavirus disease-2019 (COVID-19). The repeated emergence and outbreaks of CoVs indicate a public health threat. This suggests the possibility of animal-to-human and human-to-human transmission of newly emerging CoVs. The ongoing changes in ecology and climate make future emergence of such infections more likely [3].

## Review

Epidemiology

As of March 3, 2020, the WHO has confirmed 87,317 cases worldwide [4]. Of these confirmed cases, 2,977 (3.42%) patients have succumbed to the virus [4]. The majority of cases and deaths have been reported in China. Of the total number of cases, 79,968 (92%) patients have been identified in China [4]. Likewise, the majority of fatalities (2,873 [96.5%]) have also been reported in China [4]. It is important to note that confirmed cases are clinically diagnosed and laboratory-confirmed. Outside China, a total of 7,169 cases have emerged in 59 countries. Due to the ongoing nature of the pandemic, the number of cases and involved countries are expected to vary. Table 1 provides a comparison of the epidemiological characteristics of SARS-CoV, MERS-CoV, and SARS-CoV-2.

**Table 1:** Comparison of epidemiological characteristics between SARS-CoV, SARS-CoV-2, and MERS-CoV. SARS-CoV-2, severe acute respiratory syndrome coronavirus 2; SARS-CoV, severe acute respiratory syndrome coronavirus; MERS-CoV, Middle East respiratory syndrome coronavirus; R0, reproduction number

Features	SARS-CoV-2	SARS-CoV	MERS-CoV
Estimated R0	2.68	2-5	>1
Host of virus	Bats are natural hosts, pangolins are Intermediate hosts, and humans are terminal hosts	Chinese horseshoe bats are natural hosts, masked palm civets are intermediate hosts, and humans are terminal hosts	Bats are natural hosts, dromedary camels are intermediate hosts, and humans are terminal hosts
Transmission mode	Human-to-human through fomites, physical contact, aerosol droplets, nosocomial transmission, zoonotic transmission	Human-to-human through aerosol droplets, opportunistic airborne transmission, nosocomial transmission, fecal-oral transmission, zoonotic transmission	Respiratory transmission, zoonotic transmission, nosocomial transmission, limited human-to-human transmission, aerosol transmission
Incubation period	6.4 days (range: 0-24 days)	4.6 days	5.2 days

Etiology

CoVs are a large family of RNA viruses that are found diversely in animal species. They are known to cause diseases of the respiratory, hepatic, nervous system, and gastrointestinal systems in humans [3]. Under the electron microscope, they impart a crown-like appearance due to the presence of envelope spike glycoproteins [4]. CoVs belong to the Roniviridae, Arteriviridae, and Coronaviridae families [3]. The Coronaviridae family can be classified into four genera of alpha-COV, beta-COV, delta-COV, and gamma-COV [4]. Furthermore, beta-COV can be sub-divided into 5 lineages [5]. Gene characterization has helped identify that bats and rodents are the gene source of alpha-COV and beta-COV [4]. On the other hand, avian species are deemed as genetic sources of delta-COV and gamma-COV [4]. 

CoVs are responsible for 5-10% of acute respiratory infections [3]. It has been estimated that 2% of the population are deemed healthy carriers of these viruses [3]. Some common human CoVs include HCoV-OC43, HCoV-HKU1, HCoV-229E, and HCoV-NL63 [4]. In the immunocompetent, these CoVs clinically present with self-limiting respiratory infections and common colds [4]. In the elderly and immunocompromised, they can involve the lower respiratory tracts [4]. Other human CoVs such as MERS-CoV, SARS-CoV, and SARS-CoV-2 present with pulmonary and extra-pulmonary features [4].

SARS-CoV-2, which is responsible for the COVID-19 pandemic, is a type of beta-COV. Genomic characterization studies of the new strain have indicated an 89% nucleotide match with bat SARS-like CoVZXC21 [3,6]. There is also an 82% nucleotide match with the human SARS virus [6]. Therefore, these findings form the basis for the new strain to be called SARS-CoV-2. It has a full genomic length of 29,891 to 29,903 nucleotides. The virus is sensitive to ultraviolet light and heat [4]. SARS-CoV-2 bind to their target cells through angiotensin-converting enzyme 2 (ACE2), which is expressed in the lungs. Furthermore, these viruses can be functionally inactivated with the use of ethanol (60%), ether (75%), and chlorine-containing disinfectants.

Transmission

The initial cases were presumably linked to direct exposure to infected animals (animal-to-human transmission) at a seafood market in Wuhan, China. However, clinical cases with diversity in exposure history have emerged. This helps further elaborate that human-to-human transmission of the virus is also possible. Therefore, human-to-human transmission is now considered the main form of transmission. Individuals who remain asymptomatic could also transmit the virus [4]. However, the most common source of infection is symptomatic people. Transmission occurs from the spread of respiratory droplets through coughing or sneezing [4]. Data also suggest that close contact between individuals can also result in transmission [7]. This also indicates possible transmission in closed spaces due to elevated aerosol concentrations [4].

SARS-CoV-2 has a basic reproduction number of 2.2 [4]. This suggests that a patient can transmit the infection to two other individuals. Current data suggest that the virus has an incubation period of three to seven days [8]. These findings are based on initial cases. Therefore, further studies are needed to address transmission dynamics and incubation times. 

Clinical features

COVID-19 manifests with a wide clinical spectrum ranging from asymptomatic patients to septic shock and multiorgan dysfunction [4]. COVID-19 is classified based on the severity of the presentation [4]. The disease may be classified into mild, moderate, severe, and critical [9]. The most common symptoms of patients include fever (98.6%), fatigue (69.6%), dry cough, and diarrhea [9].

Mild Disease

Patients with mild illness may present with symptoms of an upper respiratory tract viral infection [4]. These include dry cough, mild fever, nasal congestion, sore throat, headache, muscle pain, and malaise [4]. It is also characterized by the absence of serious symptoms such as dyspnea. The majority (81%) of COVID-19 cases are mild in severity [4]. Furthermore, radiograph features are also absent in such cases [9]. Patients with mild disease can quickly deteriorate into severe or critical cases.

Moderate Disease

These patients present with respiratory symptoms of cough, shortness of breath, and tachypnea [4]. However, no signs and symptoms of severe disease are present.

Severe Disease

Patients with severe disease present with severe pneumonia. acute respiratory distress syndrome (ARDS), sepsis, or septic shock [4]. Diagnosis is clinical, and complications can be excluded with the help of radiographic studies. Clinical presentations include the presence of severe dyspnea, tachypnea (respiratory rate > 30/minute), respiratory distress, SpO2 ≤ 93%, PaO2/FiO2 < 300, and/or greater than 50% lung infiltrates within 24 to 48 hours [4]. Even in severe forms of the disease, fever can be absent or moderate [4].

In addition, 5% of patients can develop a critical disease with features of respiratory failure, RNAaemia, cardiac injury, septic shock, or multiple organ dysfunction [4,9]. Data from the Chinese Centers for Disease Control and Prevention (CDC) suggest that the case fatality rate for critical patients is 49% [4]. Patients with preexisting comorbidities have a higher case fatality rate. These comorbidities include diabetes (7.3%), respiratory disease (6.5%), cardiovascular disease (10.5%), hypertension (6%), and oncological complications (5.6%) [9]. Patients without comorbidities have a lower case fatality rate (0.9%) [9].

Acute Respiratory Distress Syndrome

The development of ARDS indicates new-onset or worsening respiratory failure. It occurs as a complication within one week of known clinical insult. The values of PaO2/FiO2 are used to distinguish ARDS based on varying degrees of hypoxia. PaO2/FiO2 ≤ 100 mm Hg is indicative of severe ARDS [4]. PaO2/FiO2 values between 100 mm Hg and 200 mm Hg are diagnostic for moderate ARDS [4]. PaO2/FiO2 values between 200 mmHg and 300 mmHg support the diagnosis of mild ARDS [4]. Levels of AST (aspartate transaminase) and ALT (alanine transaminase) at the time of admission correlate with clinical deterioration to ARDS. Therefore, higher levels at admission result in rapid clinical deterioration to ARDS.

In addition to the clinical and ventilatory criteria, chest imaging modalities such as chest X-ray, computed tomography (CT) scan, and lung ultrasound can be used to support the diagnosis. The most frequent finding on CT scan includes ground-glass opacity (86%), consolidation (29%), crazy paving (19%), bilateral disease distribution (76%), and peripheral disease distribution (33%) [10]. It is important to note that a chest X-ray has a lower sensitivity (59%) to detect subtle opacities. A CT scan can further detect mediastinal lymphadenopathy, nodules, cystic changes, and pleural effusion. The aforementioned abnormalities might be detectable before the onset of symptoms.

Sepsis and Septic Shock

Patients with COVID-19 and sepsis are deemed the most critical of them all. The accompanying multiorgan dysfunction results as a consequence of dysregulated host response to infection. Signs of organ dysfunction include severe dyspnea, low oxygen saturation, reduced urine output, tachycardia, hypotension, cold extremities, skin mottling, and altered mentation [4]. Laboratory evidence of other homeostatic dysregulation includes acidosis, high lactate, hyperbilirubinemia, thrombocytopenia, and evidence of coagulopathy [4].

Patients with septic shock are persistently hypotensive despite volume resuscitation. They may also have an accompanying serum lactate level of >2 mmol/L.

Laboratory Features

Laboratory findings specific to COVID-19 include elevated prothrombin time, LDH (lactate dehydrogenase), D-dimer, ALT, C-reactive protein (CRP), and creatine kinase [9]. In the early stages of the disease, a marked reduction in CD4 and CD8 lymphocytes can also be noted [9]. Patients in the intensive care unit have shown higher levels of interleukin (IL) 2, IL-7, IL-10, GCSF (granulocyte colony-stimulating factor), IP10 (interferon gamma-induced protein 10), MCP1 (monocyte chemotactic protein 1), MIP1A (macrophage inflammatory protein alpha), and TNF-α (tumor necrosis factor-α) [11]. They also displayed other abnormal findings indicative of coagulation activation, cellular immune deficiency, myocardial injury, renal injury, and hepatic injury [9]. In critical patients, amylase and D-dimer levels are significantly elevated [4,11]. However, blood lymphocyte counts progressively decreased [4,11]. Common to non-survivors are the elevations in ferritin, neutrophil count, D-dimer, blood urea, and creatinine levels [12]. Elevations in procalcitonin levels are not a feature of COVID-19. Therefore, an elevated level of procalcitonin may suggest an alternative diagnosis such as bacterial pneumonia. Levels of CRP correlate directly with disease severity and progression.

Diagnosis

The U.S. CDC has developed criteria for persons under investigation (PUI) [4]. If a person is deemed a PUI, immediate prevention and infection control measures are undertaken. Epidemiological factors are used to assess the requirement of testing. These include close contact with a laboratory-confirmed patient within 14 days of symptoms or travel history to an infected area within 14 days of symptom onset [4].

The WHO recommends collecting samples from both the upper and lower respiratory tracts. This can be achieved through expectorated sputum, bronchoalveolar lavage, or endotracheal aspirate [4]. These samples are then assessed for viral RNA using polymerase chain reaction (PCR). If a positive test result is achieved, it is recommended to repeat the test for re-verification purposes. A negative test with a strong clinical suspicion also warrants repeat testing.

Management

Isolation remains the most effective measure for containment of COVID-19. No specific anti-viral medication or vaccine is currently available [4]. Therefore, the treatment of COVID-19 includes symptomatic care and oxygen therapy. Patients with mild infections require early supportive management. This can be achieved with the use of acetaminophen, external cooling, oxygen therapy, nutritional supplements, and anti-bacterial therapy [9]. Critically ill patients require high flow oxygen, extracorporeal membrane oxygenation (ECMO), glucocorticoid therapy, and convalescent plasma [9]. The administration of systemic corticosteroids is not recommended to treat ARDS [4]. Moreover, unnecessary administration of antibiotics should also be avoided. ECMO should be considered in patients with refractory hypoxemia despite undergoing protective ventilation [4]. Patients with respiratory failure may require intubation, mechanical ventilation, high-flow nasal oxygen, or non-invasive ventilation [4]. Treatment of septic shock requires hemodynamic support with the administration of vasopressors. Organ function support is necessary for patients with multiple organ dysfunction [4].

Therapeutically, aerosol administration of alpha-interferon (5 million units twice daily), chloroquine phosphate, and lopinavir/ritonavir have been suggested [4]. Other suggested anti-virals include ribavirin and abidor [9]. The use of three or more anti-viral drugs simultaneously is not recommended. Ongoing clinical studies suggest that remdesivir (GS5734) can be used for prophylaxis and therapy [4]. Furthermore, a fusion inhibitor targeting the HR1 domain of spike protein is reported to have the potential to treat COVID-19.

Prevention

Preventive measures must focus on optimizing infection control protocols, self-isolation, and patient isolation during the provision of clinical care. The WHO has advised against close contact with patients, farm animals, and wild animals [4]. Patients and the general public must cover coughs and sneezes to help prevent aerosol transmission. Frequent handwashing with soap and water is also required. As an alternative measure, hand sanitizers can also be used. Immunocompromised individuals are advised to avoid public gatherings. Emergency medicine departments must apply strict hygiene measures for the control of infections. Healthcare personnel must use personal protective equipment such as N95 masks, FFP3 masks, gowns, eye protection, gloves, and gowns.

## Conclusions

The COVID-19 pandemic is spreading across the globe at an alarming rate. It has caused more infections and deaths as compared with SARS or MERS. Based on R0 values, it is deemed that SARS-CoV-2 is more infectious than SARS or MERS. Elderly and immunocompromised patients are at the greatest risk of fatality. The rapid spread of disease warrants intense surveillance and isolation protocols to prevent further transmission. No confirmed medication or vaccine has been developed. Current treatment strategies are aimed at symptomatic care and oxygen therapy. Prophylactic vaccination is required for the future prevention of COV-related epidemic or pandemic.
